# Misdiagnosis of lymphoma as vasculitis: A case report

**DOI:** 10.1002/rcr2.1347

**Published:** 2024-04-09

**Authors:** Shushan Wei, Haobin Hu, Haoyue Helena Lan, Na Li, Qingling Zhang

**Affiliations:** ^1^ Pulmonary and Critical Care Medicine, Guangzhou Institute of Respiratory Health, National Clinical Research Center for Respiratory Disease, National Center for Respiratory Medicine, State Key Laboratory of Respiratory Diseases The First Affiliated Hospital of Guangzhou Medical University Guangzhou China; ^2^ Shenzhen Clinical Medical College Guangzhou University of Chinese Medicine Shenzhen China; ^3^ Department of Family Medicine University of Toronto Toronto Ontario Canada; ^4^ Department of Pulmonary and Critical Care Medicine Shenzhen Longgang Central Hospital Shenzhen China

**Keywords:** eosinophils, lymphoma, NK/T‐cell lymphoma, vasculitis

## Abstract

NK/T‐cell lymphoma (NKTCL) is a highly aggressive malignant tumour with a very poor prognosis, which often poses diagnostic difficulties due to the non‐specificity of its clinical presentation. NK/T‐cell lymphoma with eosinophilic hyperplasia syndrome is extremely rare. This article describes a patient with NKTCL misdiagnosed as vasculitis who presented with sinusitis, abdominal pain, anorexia, and lung shadows. Additionally, the patient exhibited extremely high eosinophilia levels, which led to a further misdiagnosis of eosinophilic granuloma. We describe the clinical features, diagnostic methods and differential diagnosis of lymphoma and highlights the importance of a multidisciplinary approach in accurate diagnosis and treatment.

## Graphical abstract

This article describes a patient with NKTCL misdiagnosed as vasculitis who presented with sinusitis, abdominal pain, anorexia, and lung shadows, and also presented with eosinophilia, which was misdiagnosed as eosinophilic granuloma. The article describes the clinical features, diagnostic methods and differential diagnosis of lymphoma and highlights the importance of a multidisciplinary approach in accurate diagnosis and treatment.

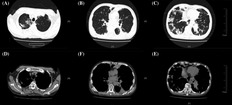

## INTRODUCTION

NK/T‐cell lymphoma (NKTCL) is frequently overlooked and misdiagnosed due to a lack of specific clinical signs.^1^ Extranodal NK/T‐cell lymphoma, nasal type (ENKTCL‐NT) is the most common type of NKTCL，which frequently builds up in the nasal cavity, sinuses, nasopharynx, and upper respiratory tract.^2^ These symptoms are frequently associated with sustained fever or weight loss, and T‐cell lymphomas with hypereosinophilic syndrome are extremely rare.^3^

## CASE REPORT

The patient is a 52‐year‐old male, who is a self‐employed merchant. He presented to hospital in September 2022 with complaints of abdominal pain, anorexia, and a lung shadow which had been present for more than 3 years. He was diagnosed with ‘allergic rhinitis and sinusitis’ 5 years ago in a local hospital; however, he didn't receive standardized treatment at that time. Between November 2018 and September 2022, he was seen by Gastroenterology at the local hospital for abdominal pain and anorexia. His peripheral eosinophil count was 3.65 × 10 9/L, with an 18.7% ratio. His gastroscopy pathology showed moderate chronic gastritis with acute activity as well as moderate atypical hyperplasia of local glandular epithelium. Additionally, eosinophils and neutrophils were present in the bloody exudate from the pancreatic head biopsy. The alveolar septa in the lung tissue were widened, while the stroma was infiltrated by lamellar lymphocytes and a significant number of eosinophils. The final discharge diagnoses were (1) Gastric lesions: eosinophilic gastritis versus Malt lymphoma versus immune gastritis. (2) Chronic pancreatitis. (3) Lung shadow—further investigation required. The patient was prescribed oral prednisone acetate (15–25 mg QD) and proton pump inhibitor for gastric protection. The progression of the patient's condition between 2018 and 2022 is shown in Table 1.

**TABLE 1:** Evolution of the patient's condition.

Time	Symptoms	Major auxiliary examinations	Pathology	Discharge diagnosis	Treatment
Nov 18, 2018	Upper abdominal pain, nocturnal dull pain	Gastroscopy: 1. Rough gastric mucosa of the fundus, nature to be determined; 2. Bile reflux gastritis; 3. Duodenal bulbitis; 4. Reflux esophagitis (LA‐A).	(Gastric mucosa biopsy) Moderate chronic gastritis, mild activity, moderate atrophy, focal glandular epithelium.	Chronic gastritis	Acid suppression, gastric protection, quadruple anti‐HP infection
May 19,2019	Abdominal pain with anorexia and weight loss	Abdominal CT and MR revealed: pancreatic morphology was full, the head was prominent, but the signal was uniform.	(Cardia, lower posterior wall of the gastric body, lower great curvature of the gastric body, lower anterior wall of the gastric body, lower minor curvature of the gastric body) Eosinophilic gastritis with erosion, partial glandular mild to moderate atypical hyperplasia.	1. Eosinophilic gastritis; 2. Autoimmune pancreatitis to be excluded.	Acid suppression, gastric protection, etc.
Aug 19, 2019	Abdominal pain with anorexia and weight loss	PET‐CT: 1. Multiple focal thickening seen in the pancreatic head, uncinate process, and body, mostly considered as pancreatitis, but the possibility of pancreatic cancer cannot be completely excluded; 2. Multiple patchy, flaky, and cord‐like shadows seen in both lungs, considered as active tuberculosis or other infectious lesions.	1. (Gastric antrum, gastric angle, gastric body, gastric fundus) Moderate chronic gastritis with acute activity, focal glandular epithelium with moderate atypical hyperplasia; 2. Bloody effusion and partial eosinophils, neutrophils, and lymphocytes seen in the pancreatic head puncture material.	1. Pancreatic mass to be investigated; 2. Chronic pancreatitis; 3. Eosinophilic gastritis.	Oral prednisone acetate (40 mg QD), acid suppression, and gastric protection
May 22, 2022	Abdominal pain, anorexia, significant weight loss (10 kg)	PET‐CT: 1. Mild thickening of the gastric wall in the antropylorus region with mild to moderate metabolic elevation, mostly considered as inflammation, but the possibility of lymphoma cannot be excluded. Multiple enlarged lymph nodes seen around the antropylorus; 2. No obvious signs of pancreatitis seen in the pancreas; 3. Multiple chronic inflammatory lesions seen in both lungs, a soft tissue density shadow seen in the posterior basal segment of the left lower lung.	(Lung tissue) Widened alveolar septa, infiltration of lymphocytes and a large number of eosinophils in the interstitium, lymphocytes with variable size and morphology, some cells with translucent cytoplasm, round, spindle, or irregular nuclei, suspected of atypia.	1. Gastric lesions: eosinophilic gastritis? Malt lymphoma? Immunogenic gastritis?; 2. Chronic pancreatitis; 3. Pulmonary shadows to be investigated	Oral prednisone acetate (15–25 mg QD) and acid suppression, gastric protection

**FIGURE 1:**
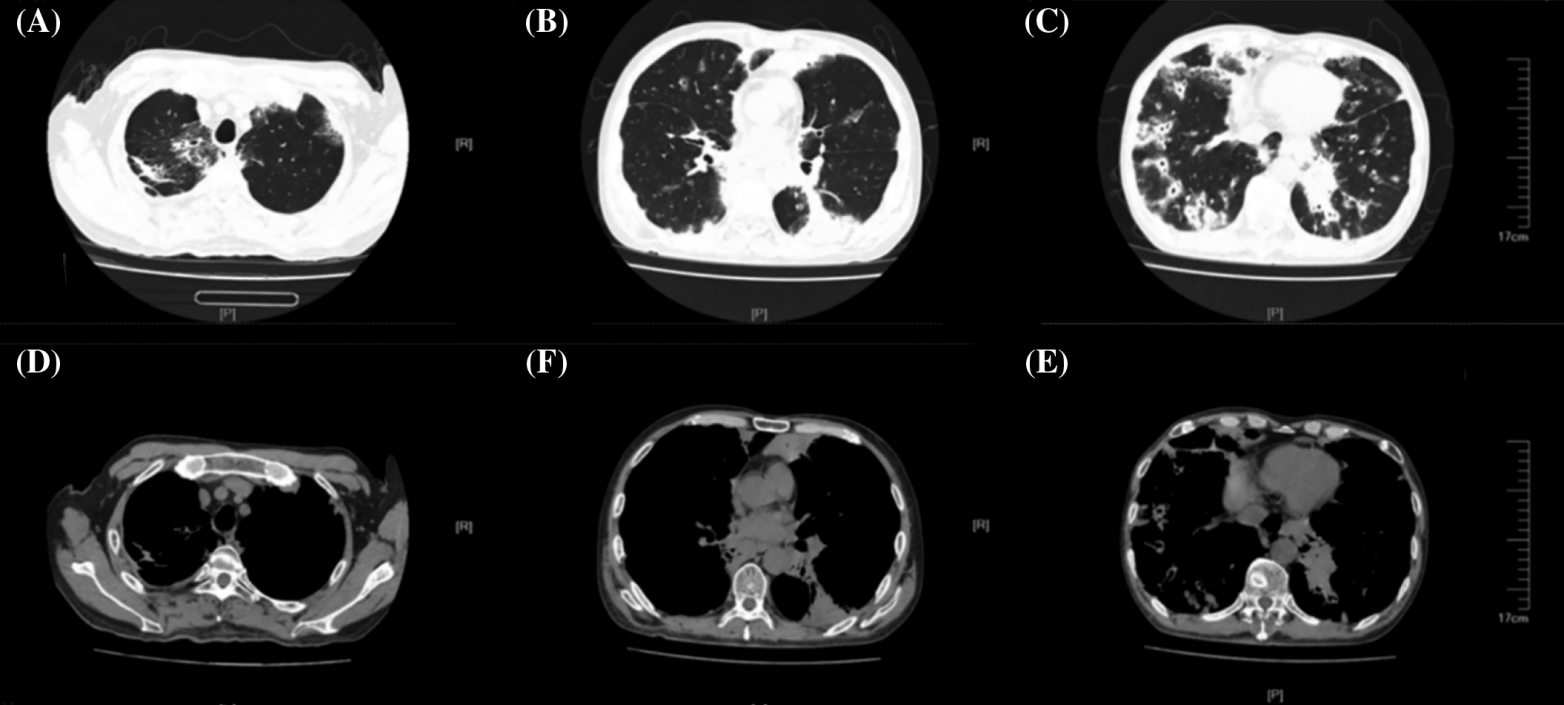
Chest computed tomography showed that multiple lesions in both lungs, suggestive of eosinophilic granulomatosis with polyangiitis (EGPA), eosinophilic pneumonia or fungal infection. Clinical correlation is necessary to make a definitive diagnosis. Multiple slightly enlarged lymph nodes in both lungs and mediastinum, suggestive of reactive hyperplasia.

**FIGURE 2:**
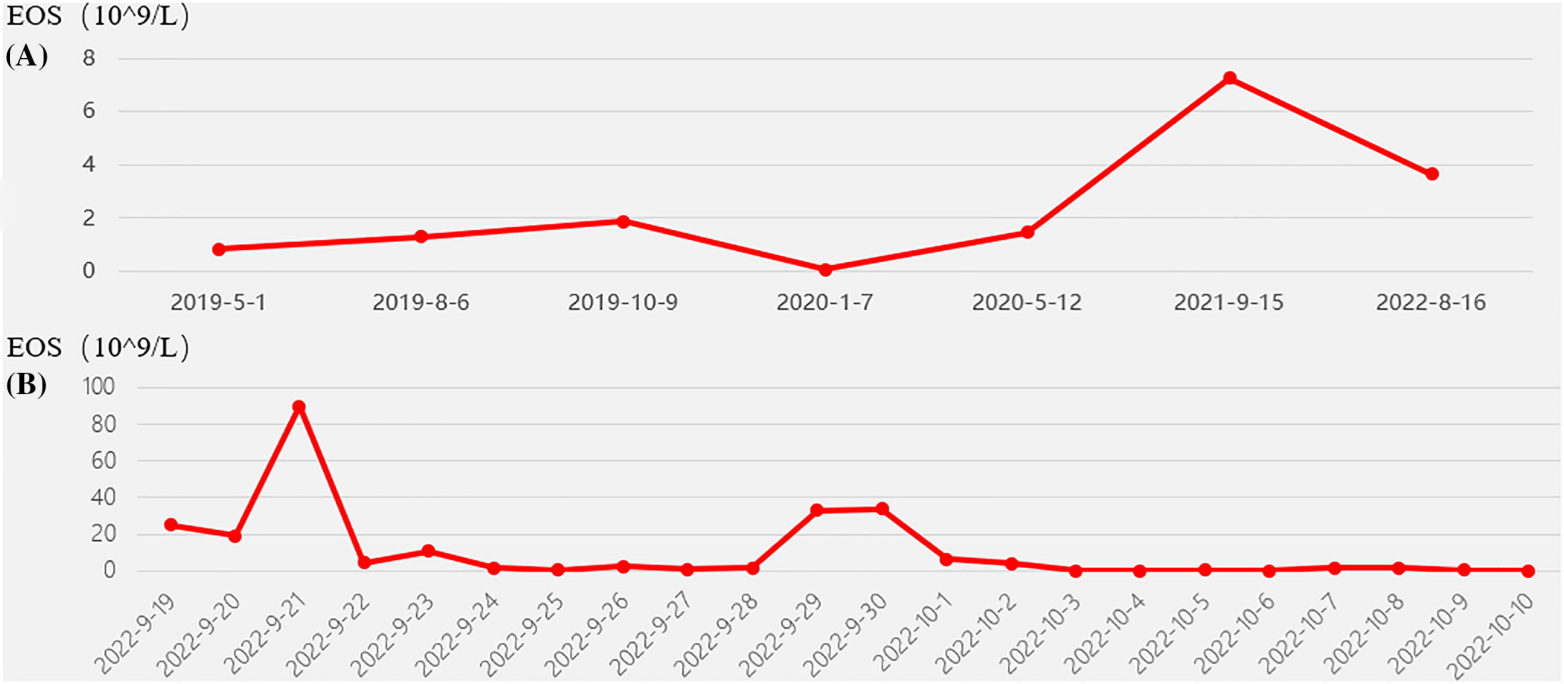
Graph of changes in eosinophil counts.

Between September 14 and October 10, 2022, he was admitted under respirology due to elevated blood eosinophils and the lung shadow (Figure 1). Upon admission, the patient's oxygen saturation was 90% on room air. He also had a scattered erythematous rash on bilateral lower limbs. The remainder of the exam was otherwise normal. Meanwhile, the eosinophil counts and trends of the patients' eosinophil counts before and after admission are shown in Figure 2.

**FIGURE 3:**
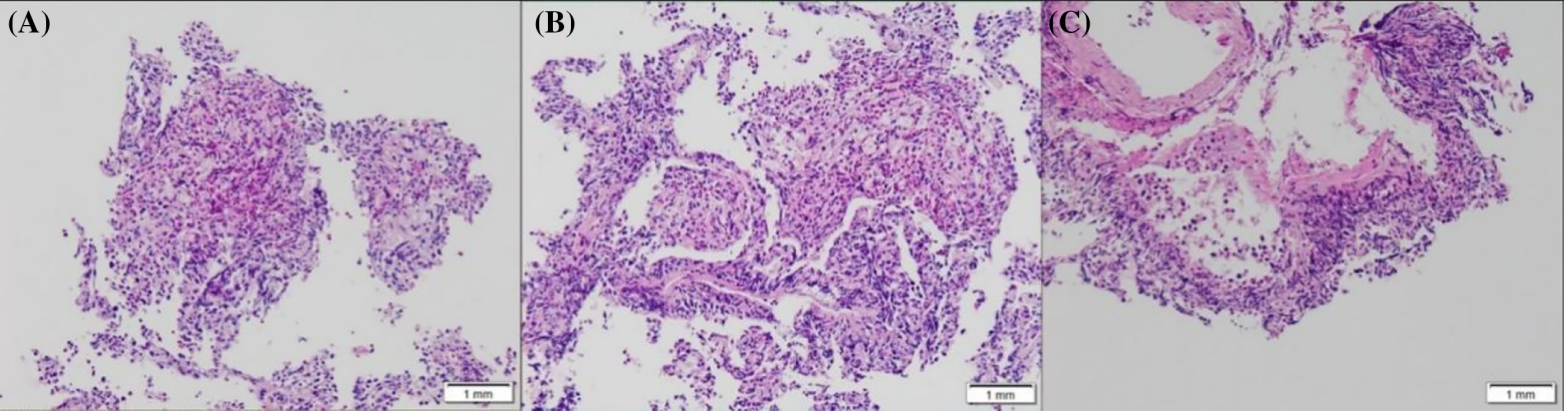
Transbronchial right lower lung biopsy: Microscopic findings: Lung tissue was sent for examination, with a large number of eosinophils, histiocytes, foamy cells in the alveolar lumen, interstitial lymphocyte infiltration, and eosinophilic infiltration of the walls of small blood vessels. Immunohistochemistry results:CD3 (+), CD5 (−), CD4 (−), CD8 (individual +), CD56 (−), CD20 (−), CD30 (+), Ki67 (30%+), in situ hybridization results: EBER (−). Special staining results: GMS (−), PAS (−), antacid (−), Gram (−), antacid fluorescence (−), fungal fluorescence (I). Pathologic diagnosis:(right lower lung) Tissue changes of eosinophilic lung disease, clinical exclusion of EGPA is recommended, immunohistochemistry of this film is insufficient to diagnose lymphoma, close follow‐up is recommended.

On September 20th, bronchoscopic biopsy of the right lower lung revealed the following microscopic findings (Figure 3): the submitted lung tissue showed abundant eosinophils, histiocytes, foamy cells in the alveolar cavity, interstitial lymphocyte infiltration, and eosinophil infiltration in the walls of small blood vessels. Immunohistochemistry results: CD3 (+), CD5 (−), CD4 (−), CD8 (individual+), CD56 (−), CD20 (−), CD30 (+), Ki67 (30%+). In situ hybridization results: EBER (−). Special staining results: GMS (−), PAS (−), acid‐fast stain (−), Gram stain (−), acid‐fast fluorescence stain (−), fungal fluorescence stain (1+). Pathological diagnosis indicated (right lower lung) tissue changes consistent with eosinophilic granulomatosis with polyangiitis (EGPA) that should be clinically excluded. There was insufficient immunohistochemistry for lymphoma diagnosis, and a was recommended. The differential diagnosis included three possibilities: (1) Eosinophilic granulomatous vasculitis (EGPA), (2) Investigation of the nature of the sinus lesions is pending and (3) Eosinophilic gastritis. Blood routine: White blood cells: 82.51× 10^9^/L; eosinophil ratio: 78.2%; eosinophil count: 64.53 × 10^9^/L; Haemoglobin: 151 g/L; Platelets: 378 × 10^9^/L; The patient was prescribed oral prednisone acetate (15–25 mg QD) and proton pump inhibitor for gastric protection.

**FIGURE 4:**
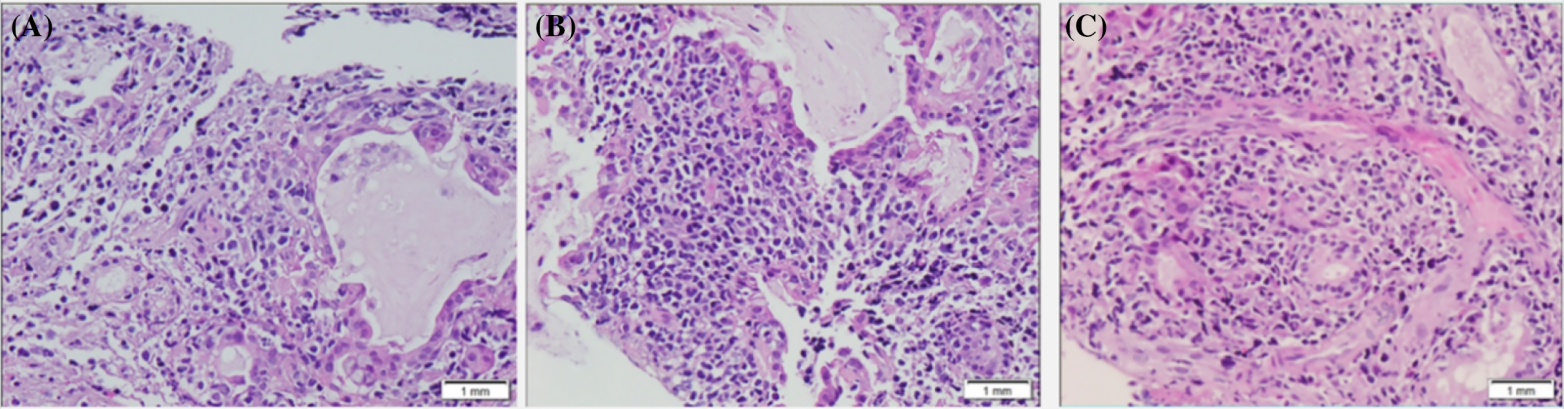
Nasal mucosal biopsy: Microscopic findings. (Lateral wall of inferior turbinate, nasal septum, lateral wall of inferior turbinate) Sent mucosal tissue, diffuse infiltration of medium to large tumour cells was seen in the submucosa, the nuclei of the tumour cells were oval, irregularly shaped, with inconspicuous nucleolus, translucent cytoplasm, invading the glandular epithelium, a small number of small lymphocytes, eosinophils, and plasma cells were seen in the background. Immunohistochemistry: CD20 (small +), CD79a (small +), CD3 (+), CD5 (−), CD4 (+), CD8 (small +), CD56 (−), CD2 (+), Ki67 (40% +), CD10 (+), CD21 (−), CD23 (small +), CXCL‐13 (−), CK (−). In situ hybridisation: EBER (−). Pathological diagnosis. (lateral wall of inferior turbinate, nasal septum, lateral wall of inferior turbinate) Tissue changes as non‐Hodgkin's lymphoma with a tendency towards non‐specific peripheral T‐cell lymphoma.

After admission, the patient developed shortness of breath, intermittent fever, abdominal pain. After a multidisciplinary discussion, there was strong suspicion for systemic diseases that affect multiple organs, and eosinophilic granulomatous vasculitis could not be ruled out. The patient received treatment consisting of hydroxyurea, imatinib, cyclophosphamide, and methylprednisolone. However, despite these treatments, the patient's respiratory failure gradually worsened. After high flow nasal canula (FiO_2_ 60%) support was utilized, his SpO_2_ fluctuated between 90% and 95%, with a P/F ratio less than 150. Between October 10 and 27, 2022, he was transferred to the intensive care unit. A right nasal mucosa biopsy was conducted on October 20, 2022. Microscopically, (lateral wall of inferior turbinate, nasal septum, and lateral wall of inferior turbinate) sent mucosal tissue, diffuse infiltration of medium to large tumour cells was seen in the submucosa, the nuclei of the tumour cells were oval, irregularly shaped, with inconspicuous nucleolus, translucent cytoplasm, invading the glandular epithelium, a small number of small lymphocytes, eosinophils, and plasma cells were seen in the background. Immunohistochemical results were as follows (Figure 4): CD20 (small +), CD79a (small +), CD3 (+), CD5 (−), CD4 (+), CD8 (small +), CD56 (−), CD2 (+), Ki67 (40% +), CD10 (+), CD21 (−), CD23 (small +), CXCL‐13 (−), and CK (−). In situ hybridisation: EBER (−). Pathology indicated changes in the lateral wall of the inferior turbinate, nasal septum, and lateral wall of the inferior turbinate, and the results revealed non‐Hodgkin's lymphoma with a tendency towards nonspecific peripheral T‐cell lymphoma. He was treated with Dilizumab and Cedaramine. The final diagnosis was: non‐Hodgkin's lymphoma with a tendency towards nonspecific peripheral T‐cell lymphoma. Not long after receiving treatment, the patient voluntarily signed off treatment and was discharged due to financial reasons. After follow‐up, the patient eventually died of septic shock.

## DISCUSSION

NK/T‐cell lymphoma is a subtype of non‐Hodgkin's lymphoma that primarily affects extra‐nodal organs. Its pathogenesis is not yet fully understood, but it is commonly associated with EBV infection and chronic rhinitis. Other factors, such as genetics and long‐term exposure to radiation, may also contribute to its development.^1^ The lesions in most patients are concentrated in the midline region of the face, particularly in the nasal cavity, and are characterized by disfiguring features. NKTCL is a highly aggressive lymphoma that can rapidly spread to other extranodal sites. Lung involvement can present with various manifestations, such as solid lesions, multiple nodules, and ground‐glass shadows, which can be misdiagnosed as pneumonia.^2^, ^3^ NK/T‐cell lymphoma with eosinophilia syndrome is a rare condition that can be easily misdiagnosed due to atypical clinical symptoms.

The gold standard for diagnosing NKTCL is histopathological biopsy and immunohistochemical examination. Morphologically, NKTCL is characterized by coagulative necrosis and mixed infiltration of various inflammatory cells, accompanied by prominent proliferation of atypical cells. Tumour cells vary in size and morphology, ranging from small to large cells or anaplastic cells. Tumour cells invade the walls of small blood vessels and surrounding tissues, with fibrinoid necrosis and vasculitis visible in small vessels. The immunophenotype primarily expresses CD56 and CD2, with positivity for cytotoxic markers such as TIA‐1, perforin, and granzyme B, while the majority of cells are EBV‐positive.^4^

In clinical practice, NKTCL generally needs to be distinguished from the following diseases: eosinophilic granulomatous polyangiitis (EGPA), chronic sinusitis and lymphomatoid granulomatosis. EGPA is a systemic small and medium‐sized vessel necrotizing vasculitis associated with antineutrophil cytoplasmic antibodies (ANCA), which can be accompanied by eosinophil infiltration into tissues. Patients may have a history of asthma, allergic rhinitis, nasal polyps, and so on, and their symptoms can improve after immunosuppressive treatment. Histopathological and immunohistochemical examination are the main methods for differential diagnosis.^5^ Chronic sinusitis clinical manifestations and imaging findings are non‐specific, similar to the early manifestations of ENKTCL‐NT. However, most patients can alleviate their symptoms after treatment with nasal drops and antibiotics. Generally, the nasal mucosa of patients is smooth without extensive erosion or scabs. Lymphomatoid granulomatosis is a vascular‐centered and vascular‐destructive lymphoproliferative disease that preferentially affects extra‐nodal sites. It is composed of EB virus‐infected B cells mixed with a large number of reactive T cells, and necrosis is common.^6^

The patient was diagnosed with vasculitis based on early pathological indications of inflammatory lesions, atypical early imaging manifestations, and elevated eosinophils with multisystemic damage. Although the patient did not present with asthma or sinus lesions, the presence of markedly elevated peripheral blood eosinophils, eosinophil infiltration of lung tissue and its small vessel walls, and multisystemic damage including skin rash strongly suggested EGPA.^7^ This presentation is very rare in patients with NKTCL, so the diagnosis was finally confirmed through pathological biopsy after treatment for vasculitis was ineffective. The cellular morphology of NK/T‐cell lymphoma tumour cells is diverse. Tumour necrosis leads to an inflammatory reaction, resulting in the centre of the lesion being mostly necrotic tissue with reactive inflammatory cells. The background of numerous reactive cells can easily blur the infiltration of tumour cells. Diagnosing ENKTCL‐NT pathology can be challenging due to small and brittle biopsy samples, few tumour cells, and large necrotic areas. To improve the accuracy of the biopsy, it is recommended to take the biopsy site at the junction of the necrotic foci and the diseased tissues. If necessary, the biopsy should be repeated several times and taken at multiple points. Additionally, the tissue block should be large enough.

The diagnosis, treatment, and learning process of this case have highlighted the importance of early MDT (multidisciplinary team) discussion for diseases with unknown aetiology and multi‐system damage. This approach allows for a comprehensive search for the aetiology from multiple perspectives, enabling precise treatment.

## AUTHOR CONTRIBUTIONS

Shushan Wei wrote and approved the manuscript. Haobin Hu wrote and approved the manuscript. Haoyue Helena Lan wrote and approved the manuscript. Na Li conceived, wrote, and approved the manuscript. All authors read and approved the final manuscript.

## FUNDING INFORMATION

Funded by the Construction of Key Medical Disciplines in Longgang District, Shenzhen.

## CONFLICT OF INTEREST STATEMENT

None declared.

## ETHICS STATEMENT

Ethics approval and consent to participate.

## CONSENT FOR PUBLICATION

All authors have read and approved the final manuscript.

## Data Availability

The data that support the findings of this study is available on request from the corresponding author. The data are not publicly available due to privacy restriction.
